# Assessment of nutrient intake by gut microbiota enterotype in Japanese subjects

**DOI:** 10.1080/29933935.2026.2705623

**Published:** 2026-07-30

**Authors:** Tomohisa Takagi, Ryo Inoue, Akira Ohshima, Machi Oda, Hiroshi Sakazume, Kenta Ogawa, Tomohide Tanaka, Tomo Tominaga, Yoichi Mihara, Norihiro Ouchi, Atsuo Adachi, Tadaaki Kamitani, Satoaki Matoba, Jin Narumoto, Katsura Mizushima, Takeshi Yasuda, Hiroaki Kitae, Kazuhiko Uchiyama, Yuji Naito

**Affiliations:** a Molecular Gastroenterology and Hepatology, Graduate School of Medical Science, Kyoto Prefectural University of Medicine, Kyoto, Japan; b Laboratory of Animal Science, Setsunan University, Osaka, Japan; c Department of Research and Development, PreMedica Inc, Tokyo, Japan; d Department of Internal Medicine, Kyotango Municipal Yasaka Hospital, Kyoto, Japan; e Department of Cardiovascular Medicine, Graduate School of Medical Science, Kyoto Prefectural University of Medicine, Kyoto, Japan; f Department of Psychiatry, Graduate School of Medical Science, Kyoto Prefectural University of Medicine, Kyoto, Japan; g Department of Human Immunology and Nutrition Science, Graduate School of Medical Science, Kyoto Prefectural University of Medicine, Kyoto, Japan

**Keywords:** Gut microbiota, enterotypes, dietary patterns, nutrient intake, Japanese population

## Abstract

Japanese gut microbiota forms community clusters and shares some compositional features with Western enterotypes, although the relationship between enterotypes and habitual nutrient intake in Japanese adults remains unclear. We analyzed data from 770 community-dwelling adults. Enterotypes were assigned based on 16S rRNA gene profiles using a support vector machine model, and taxonomic annotations were updated using the SILVA 138 database. Dietary intake and nutrient intake were assessed, and differences among enterotypes were examined. Candidate nutrients and food groups potentially associated with enterotypes were prioritized using a SHapley Additive explanation (SHAP)-based interpretation of the XGBoost models and were further evaluated using linear discriminant analysis. Five enterotypes (Types A–E) were identified and exhibited insufficient dietary fiber and excessive salt intake. Types A and C showed the highest and lowest overall nutrient intake scores, respectively. Although predictive performance for nutrient intake and food-group intake was modest, linear discriminant analysis based on SHAP-prioritized features revealed distinct but partially overlapping dietary signatures among enterotypes. Correlation analyses identified associations between key bacterial taxa and enterotype-specific dietary features, which reflect complex combinations of nutrients and food groups. These findings may contribute to the development of enterotype-aware dietary strategies to promote a healthy gut microbiota.

## Graphical abstract

**Figure:**
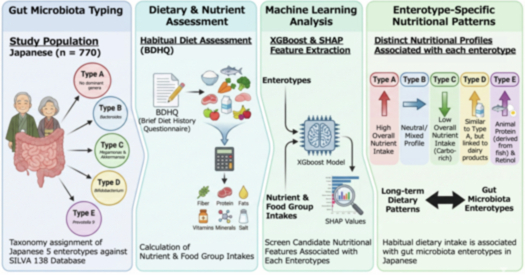

## Introduction

The gut microbiota plays a pivotal role in the metabolic, physiological, and immunological systems of humans,^1^ and its structure is closely associated with an individual’s health and disease status. The gut microbiota varies greatly between individuals, making it challenging to interpret its relationship with health and disease. To address this complexity, the concept of “enterotypes” has been proposed as a framework that clusters individuals into distinct community types based on shared microbial composition, thereby facilitating a more structured understanding of gut microbiome variation. Arumugam et al. first demonstrated that the gut microbial community structures of humans can be classified into three enterotypes, each of which is defined by a high abundance of *Bacteroides*, *Prevotella*, and *Ruminococcus.* ^2^ Later, using a larger cohort, Holms et al. stratified the gut microbiota into four enterotypes: *Ruminococcaceae* (R), *Prevotella* (*P*), *Bacteroides 1* (B1), and *Bacteroides 2* (B2) enterotypes.^3^ The B2 enterotype is characterized by a lower proportion of *Faecalibacterium* compared with the B1 enterotype.

Although clustering of the human gut microbiota using Western cohorts is useful in assessing associations with various diseases and in evaluating alterations in the gut microbiota resulting from therapeutic interventions, it is also well known that the composition and structure of the gut microbiota differ among region and ethnic groups. Nishijima et al. confirmed that the gut microbiome of the Japanese population differs considerably from that of other populations.^4^ Therefore, we previously demonstrated the enterotypes of Japanese gut microbiota communities, and the Japanese gut microbiota was stratified with good reproducibility into five community types, unlike the enterotypes proposed for individuals from Western countries, and we also attempted to assess the association of various diseases with the gut microbiota community typing.^5^ We reconfirmed that the Japanese gut microbiota was especially characterized by the presence of the genus *Bifidobacterum*, and while some enterotypes were identified as relatively healthy with limited association with diseases, other clusters showed strong associations with various conditions, including lifestyle-related diseases such as dyslipidemia, diabetes, and inflammatory bowel disease.

Given that diet is one of the major ecological forces shaping gut microbial community structures, enterotypes are associated with long-term dietary patterns. Dietary components, such as carbohydrates, proteins, fats, fibers, polyphenols, and vitamins, can affect the abundance and diversity of gut microbes as well as their metabolic activities and interactions.^6^ For instance, *Prevotella* is strongly associated with a carbohydrate-rich diet, whereas the *Bacteroides* enterotype is associated with protein and animal fat consumption, which is common in Western diets.^7^ Additionally, Western dietary patterns have been associated with lower levels of genes related to short-chain fatty acid synthesis, amino acid metabolism, and bile acid transformation in the gut microbiome.^6^

However, despite the established importance of diet in shaping gut microbial communities, the association between dietary, nutrient intake, and the five Japanese enterotypes remains unclear. In the present study, we investigated the association between each enterotype and nutritional habits based on a detailed nutritional survey of older Japanese cohorts.

## Materials and methods

### Study design and participants

This study was a cross-sectional analysis conducted as part of the Kyotango Multi-purpose Cohort Study, initiated by the Department of Longevity and Community Epidemiology at Kyoto Prefectural University of Medicine. It involved 800 community-dwelling residents aged ≥ 65 years from the Kyotango area in northern Kyoto Prefecture who had available gut microbiota information and dietary and nutrient intake data. The Kyotango region, comprising two cities and two towns, has a population of just under 100,000, with over 38% of residents aged 65 years or older, making it one of the most aged regions in Kyoto Prefecture. Despite its aging population, the region is known for its high number of centenarians, with more than 200 per 100,000 individuals, making it a notable area for longevity in Japan.

The flow of the analysis is shown in Supplementary Figure 1.

### Classification of Japanese enterotypes

The microbial community structure, namely the Japanese enterotypes of each individual, was classified using a support vector machine-based classification method defined in our previous report.^5^ As this support vector machine model relies on the Greengenes taxonomic database (GG1), which is outdated, we verified the bacterial taxonomic differences among enterotypes using the SILVA 138 database. Original fastq files were processed, taxonomy assignment was performed as described by Miura et al.,^8^ and the version of QIIME2 version 2024.5 was used.

To assess the differences in the relative abundances of major bacterial genera among enterotypes, the relative abundances of bacterial genera (assigned against the SILVA database) were filtered to retain taxa with a mean abundance of at least 0.5% across all samples. Differences in relative abundance among enterotypes were assessed using the Kruskal–Wallis (KW) test. *P*-values were adjusted for multiple testing using the Benjamini–Hochberg (BH) method.

### Assessment of dietary and nutrient intakes

A Brief-type Self-administered Diet History Questionnaire (BDHQ) was used to assess dietary and nutrient intakes.^9^ Samples with an energy intake exceeding 3,000 kcal and a body mass index (BMI) less than 30 were excluded, leaving 770 participants for analysis after the exclusion of 30 individuals. The excluded BDHQ data were considered abnormal for older individuals and were suspected to result from inaccurate responses to the BDHQ.

The intake of nutrients and food groups was normalized to the energy intake and expressed as amounts per 1,000 kcal. For each nutrient, the differences among enterotypes were assessed using the KW test. *P*-values were adjusted for multiple tests using the BH method. The same approach was applied to dietary intake, separately from nutrient intake.

### Calculation of nutrition intake scores

We first evaluated the nutrient intake scores derived from the dietary records to enable approximate profiling of the dietary composition of individuals. Reference values for 28 nutrients corresponding to participants’ age and sex were obtained from the Dietary Reference Intakes for Japanese (2025 edition).^10^ In addition to these nutrients, reference values for sucrose intake (<5% of energy) were added based on World Health Organization recommendations. For each nutrient, an individual score was calculated based on how closely the intake aligned with the corresponding dietary reference value. For nutrients classified under “adequacy” (e.g., recommended or adequate intake), the score was calculated as the ratio of intake to target value, capped at 1.0. For “moderation” nutrients with an upper limit, participants received a full score if their intake was less than or equal to the threshold; otherwise, the score was penalized in proportion to the extent of excess. For nutrients defined by the range of intake, such as the Acceptable Macronutrient Distribution Range, a full score was assigned if the intake was within a specified range. Outside this range, the score decreased linearly based on the distance from the nearest boundary. The total nutrient score was the sum of all nutrient scores (maximum 29), while the macronutrient score was the sum of the protein, carbohydrate, and lipid scores (maximum 3).

### Extraction of nutritional and dietary features associated with enterotype structure by SHapley Additive exPlanation (SHAP) based on XGBoost

To identify nutrients and food groups associated with enterotype structure, we performed SHapley Additive explanation (SHAP) analyzes based on extreme gradient boosting (XGBoost).

Among the full set of nutrient variables, highly collinear variables (absolute Pearson correlation ≥ 0.9) were grouped, and one representative variable from each group was retained. This minimized redundancy and mitigated the impact of multicollinearity, thereby allowing for the construction of a more parsimonious and interpretable model. Accordingly, 26 representative nutrients were selected for the present study (Table 1). In parallel, food-group level variables (dietary intake) were analyzed using the same procedure, but no variables were correlated in this case. For both datasets, the intake values were normalized per 1,000 kcal of total energy intake and standardized (z-score normalization).

**Table 1.** List of representative nutrients and its correlated nutrients.

Representative nutrients	Correlated nutrients								
*α*-Carotene									
*α*-Tocopherol									
Alcohol									
Animal Fat	C14:0(S)	C15:0(S)	C16:0(S)	C16:1(M)	C17:0(S)	C17:1(M)	C18:0(S)	C18:1(M)	C20:0(S)
	C20:2(n6)(P(*n*-6))	C20:3(n6)(P(*n*-6))	C20:4(n6)(P(*n*-6))	C22_4N6	C24:0(S)	Fat	Monounsaturated fatty acids	Saturated fatty acids	
Animal protein	Niacin	Phosphorus	Protein						
*β*-Carotene	*β*-Carotene equivalent								
Plant Fat	C18:2(n6)(P(*n*-6))	C18:3(n3)(P(*n*-3))	C18:3(n6)(P(*n*-6))	C22:0(S)	Polyunsaturated fatty acids	*β*-Tocopherol	*γ*-Tocopherol	*n*-6 Fatty acids	
*n*-3 Fatty acids	C16:3(?)(P(*n*-6))	C16_2	C16_4	C18:4(n3)(P(*n*-3))	C20:1(M)	C20:4(n3)(P(*n*-3))	C20:5(n3)(P(*n*-3))	C21_5N3	C22:1(M)
	C22:5(n3)(P(*n*-3))	C22:5(n6)(P(*n*-6))	C22:6(n3)(P(*n*-3))	C24:1(M)					
C12:0(S)	C4:0(S)	C10:0(S)	C10:1(M)	C13_0	C14:1(M)	C15_0A	C16_0I	C17_0A	C7_0
	C6:0(S)	C8:0(S)							
Calcium									
Carbohydrate									
Cholesterol									
Copper									
Cryptoxanthin									
*δ*-Tocopherol									
Daidzein	Genistein								
Folic acid	Potassium								
Manganese									
Plant protein									
Retinol equivalent	Retinol								
Salt equivalent	Ash	Iron	Magnesium	Pantothenic acid	Sodium	Vitamin B1	Vitamin B2	Vitamin B6	Zinc
Sucrose									
Total dietary fiber	Insoluble dietary fiber	Water-soluble dietary fiber							
Vitamin B12	Vitamin D								
Vitamin C									
Vitamin K									

To minimize redundancy and mitigate the impact of multicollinearity, thereby allowing the construction of a more parsimonious and interpretable model, highly collinear nutrients (absolute Pearson correlation ≥ 0.9) were grouped, and one representative nutrient from each group was retained.

An XGBoost classifier was implemented in R (XGBoost package), and the SHAP values were computed using the fastshap package. To address class imbalance, the Synthetic Minority Over-sampling Technique (SMOTE, smotefamily package) was applied only within training folds during model training. For enterotypes with fewer than 200 samples, synthetic samples were generated until the class size reached 200; for those with over 200 samples, random undersampling was applied to reduce the size to 200. This yielded a balanced dataset of 200 samples per enterotype within each training fold.

Model training was repeated 50 times with different random seeds. In each iteration, the original dataset was randomly divided into training and independent test sets. Within the training set, 5-fold cross-validation was performed to optimize the number of boosting rounds. During cross-validation, scaling and SMOTE were applied exclusively to the training folds, whereas validation folds remained untouched except for scaling parameters derived from the corresponding training folds. The main model parameters were as follows: learning rate (eta) = 0.05, maximum tree depth = 3, subsample = 0.7, colsample_bytree = 0.9, min_child_weight = 5, and gamma = 1. The model performance was evaluated based on the overall classification accuracy and Cohen’s kappa using the caret package.

For each iteration, Monte Carlo simulations were applied to estimate the SHAP values for each nutrient (or dietary variable) using independent test samples not used during model training. Absolute SHAP values were statistically tested against zero using the Wilcoxon signed-rank test, followed by false discovery rate (FDR) adjustment (BH method). Nutrients (or dietary variables) with adjusted *P*-values ≤ 0.05 were regarded as significant in that iteration. The frequency of significance across 50 iterations (SigFreq) and the frequency with which each feature appeared among the top five features ranked by mean absolute SHAP values within each iteration (RankFreq) were calculated to prioritize reproducible candidate features associated with enterotype structure.

### Validation of extracted nutritional and dietary features using linear discriminant analysis (LDA)

To further evaluate whether the prioritized candidate features collectively captured enterotype-associated dietary patterns, linear discriminant analysis (LDA) was conducted separately on the nutrient and dietary intake data.

For each enterotype, candidate features were selected based on SHAP-derived stability metrics. Features were retained if they showed either a significance frequency across 50 iterations (SigFreq) ≥ 0.2, or a SigFreq ≥ 0.1 together with a high appearance frequency among the top-ranked SHAP features within each enterotype (RankFreq within the top 10).

To account for sample size imbalance, we randomly selected 30 individuals per enterotype. Each enterotype was then compared to all other enterotypes combined (one-vs. -rest classification).

The LDA models were fitted using the extracted features, and group separation was evaluated along the first discriminant axis (LD1). Statistical significance of group separation was assessed using the Wilcoxon rank-sum test. The effect size (r) was also calculated using the Wilcoxon test statistics to quantify the magnitude of group separation.

### Correlation analysis

To evaluate pairwise associations between nutrient/food-group intake and the relative abundance of major bacterial genera (see above), Spearman’s rank correlation coefficients were calculated. All correlation *P*-values were adjusted using the BH method to control the FDR.

### Data analysis software

All analyzes were performed using R (version 4.4.2) and RStudio (version 2024.12.0), unless otherwise stated. We used the following packages for data analysis and visualization: tidyverse^11^ for data manipulation; FSA and coin^12^ for non-parametric tests, ggplot2^13^ and ggpubr for data visualization; pheatmap for heatmap generation; fastshap^14^ and XGBoost^15^ for machine learning and SHAP value estimation; smotefamily and caret^16^ for class balancing and model training, MASS^17^ for LDA; and fmsb for radar chart generation.

### Ethics statements

The study conformed to the ethical principles of the Declaration of Helsinki, and the research protocol was approved by the Ethics Committee of Kyoto Prefectural University of Medicine (approval numbers: ERB-C-885-9 and ERB-C-2182-1). All participants provided written informed consent prior to enrollment. This study was registered at the University Hospital Medical Information Network Center (UMIN000045216).

## Results

### Enrolled study participants

**Figure 1.**
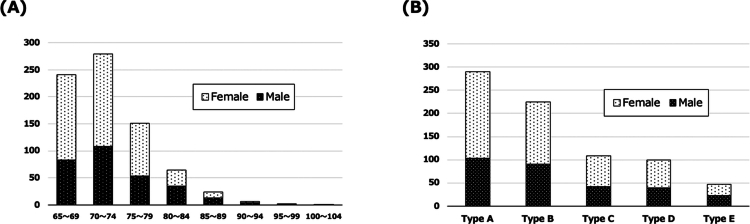
Distribution of study participants and gut microbiota enterotype. (A) Age group distribution of study participants stratified by gender. (B) Distribution of five gut microbiota enterotypes (Types A–E) stratified by gender.

We enrolled 770 participants (299 males and 471 females), as shown in Figure 1A. The average age of all participants was 72.9 ± 5.8 years (range: 65–101 years). Most participants were between 65 and 79 years of age, with a higher proportion of females across most age groups.

The enterotype classification based on the gut microbiota profiles of the enrolled participants is illustrated in Figure 1B; Type A was the most prevalent. The physical characteristics of each enterotype are shown in Supplementary Table 1. Age was significantly higher in Type A, body weight was significantly higher in Type C than in Types A and D, and BMI was significantly higher in Type C than in Type D.

### Bacterial taxonomic features of enterotypes

**Figure 2.**
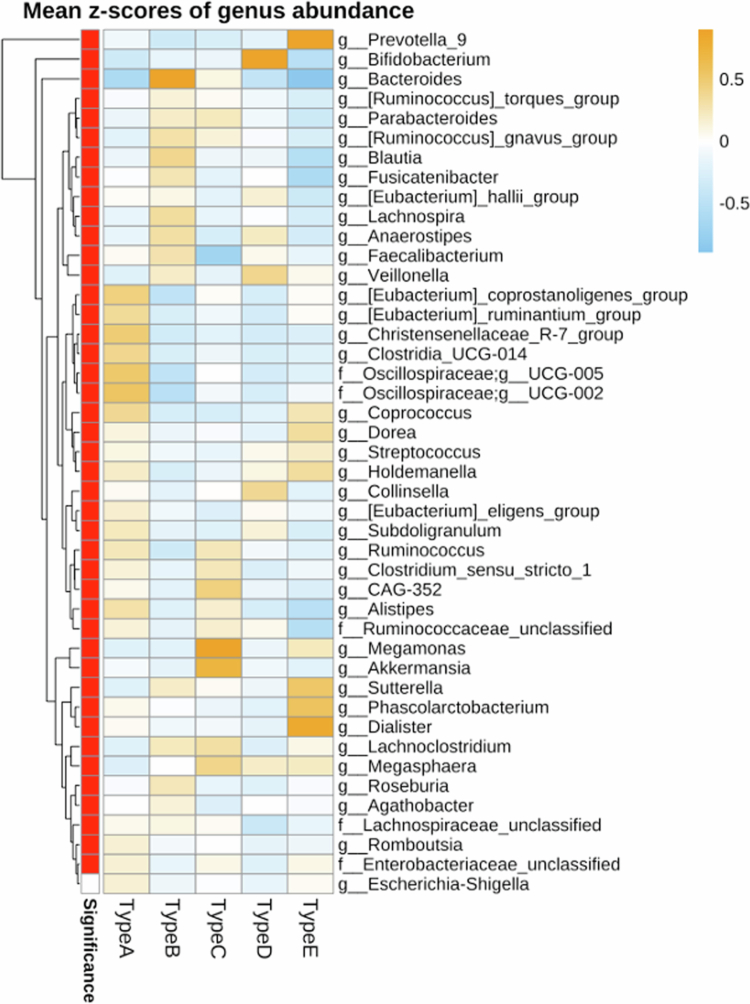
Mean standardized (z-score) abundances of bacterial genera across enterotypes. The heatmap shows the mean z-scores of the relative abundances of the bacterial genera for each enterotype. Rows represent bacterial genera, and columns represent enterotypes (Types A–E). For each genus, the relative abundance values were standardized (z-score transformation) within the cohort, and the mean values were calculated for each enterotype. Only major genera with a mean relative abundance ≥ 0.5% across all samples were included in the analysis. Statistical significance among enterotypes was evaluated using the KW test, followed by Dunn’s tests with BH correction for multiple testing. Row-side annotations indicate the level of significance based on FDR-adjusted *P*-values: red = Strong (FDR ≤ 0.1), orange = Moderate (0.1 < FDR ≤ 0.2), white = Not significant (FDR > 0.2). The color intensity in the heatmap represents the standardized abundance, ranging from blue (low) to orange (high). Genera were clustered based on similarities in abundance patterns across enterotypes (Euclidean distance).

The distribution of the relative abundances of the major genera is shown in Figure 2. The characteristic taxonomic features remained consistent with those reported previously, even when using a newer database.^7^ However, analysis using the updated database revealed additional genus-level characteristics for each enterotype.

While Types B to E showed a single or a couple of dominant genera, Type A did not and had several predominant genera, such as *Oscillospiraceae* UCG-002, *Oscillospiraceae* UCG-005, and the *Eubacterium coprostanoligenes* groups; all of them were classified as *Ruminococcaceae* in GG1. The dominant genera in Type B was *Bacteroides,* and that in Type D was *Bifidobacterium*. Types C and E showed two dominant genera, *Megamonas* and *Akkermansia* for the former, and *Prevotella* 9 and *Dialister* for the latter. These taxa are regarded as key taxa for discriminating enterotypes.

### Habitual nutrient intakes per enterotype

To evaluate the nutrient intake of each enterotype, we used the algorithm described in the Materials and Methods section. Importantly, the dietary fiber intake was insufficient across all enterotypes, highlighting the well-recognized issue of inadequate dietary fiber intake in the Japanese population (Supplementary Figure 2). Additionally, a high proportion of participants showed insufficient intake of minerals, such as calcium and magnesium, and vitamins, including vitamin B1. In contrast, excessive salt intake was evident across all enterotypes.

**Figure 3.**
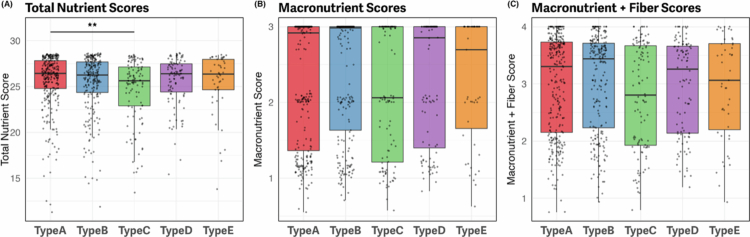
Boxplots show the distributions of nutrient intake scores among the enterotypes. Each score was calculated based on the adequacy of the individual nutrient intake relative to the Japanese Dietary Reference Intakes (2025 edition). Higher scores indicate greater adherence to the recommended intake range. Statistical differences among enterotypes were evaluated using the KW test. When the KW test was significant (*p* < 0.05), post-hoc Dunn’s tests with BH correction were conducted. Significant pairwise differences are indicated by asterisks above the boxes (* *P* < 0.1, ** *P* < 0.05). (A) Total nutrient score (sum of the adequacy scores for 29 nutrients). (B) Macronutrient scores (proteins, fats, and carbohydrates). (C) Macronutrient and dietary fiber scores.

Figure 3 illustrates the enterotype classification, with the Y-axis indicating nutrition intake scores. The same trend was observed across all scores: Types A and B exhibited a higher level of nutrient fulfillment, whereas Type C was characterized by a substantially lower score. Thus, Type C showed a tendency toward lower nutrient intake than the other enterotypes.

**Figure 4.**
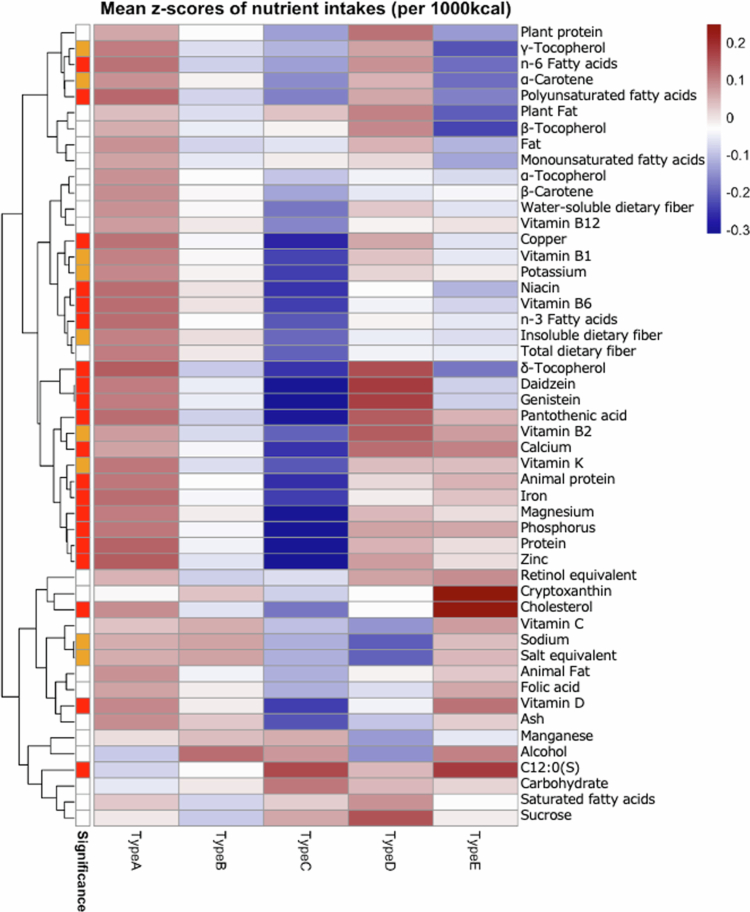
Mean standardized (z-score) nutrient intakes per 1,000 kcal across enterotypes. The heatmap shows the mean z-scores of nutrient intake normalized per 1,000 kcal for each enterotype (Types A–E). Rows represent nutrients, and columns represent enterotypes. For each nutrient, individual intake was standardized (z-score transformation) within the cohort, and the mean values were calculated by enterotype. Differences in nutrient intake among the enterotypes were evaluated shown in Figure 2. For row-side annotations, see the legend to Figure 2. The color intensity in the heatmap represents the standardized intake, ranging from dark blue (low) to dark red (high).

Differences in the mean intake of nutrients and food groups among the enterotypes are shown in Figure 4 and Supplementary Figure 3, respectively. Each enterotype exhibited a characteristic nutrient intake profile. The intake of most nutrients was higher in Type A and lower in Type C. The profile of Type D was similar to that of Type A. The profile of Type B was generally neutral compared with that of the other types, while that of Type E was heterogeneous. For macronutrients, differences in the intake of protein and animal proteins were observed among the enterotypes. Significant differences among the enterotypes were found in the intake of fatty acids and minerals (Figure 4).

Regarding food-group intake, the profiles were also characteristic of each enterotype. However, the KW test for each diet with BH adjustment demonstrated significant differences only in fish with bones, low-fat milk, and mushrooms (Supplementary Figure 3).

### Nutrient and dietary features associated with enterotype classification

To assess whether habitual dietary features were associated with enterotype classification, we constructed predictive models using XGBoost and evaluated feature contributions with SHAP values. Separate models were developed for nutrient and food-group intake. Model performance was first assessed to confirm predictive accuracy, followed by interpretation of the most influential features as determined by the SHAP values.

For the nutrient-based model, modest and consistent discriminative performance was observed across 50 repeated analyzes, with a mean test accuracy of 0.25 and a mean Cohen’s kappa of 0.05. Similarly, the food-group intake-based models achieved a mean test accuracy of 0.27 and a mean Cohen’s kappa of 0.02 across 50 repetitions. Although predictive performance was limited, several dietary features were repeatedly prioritized across repeated analyzes based on SHAP-derived stability metrics, suggesting the presence of reproducible but overlapping dietary signatures associated with enterotype structure.

**Figure 5.**
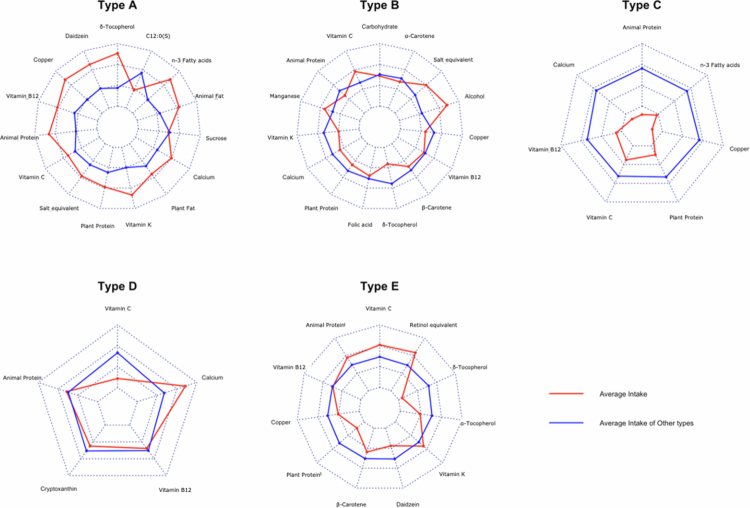
Radar plots of SHAP-extracted nutrient intake profiles by enterotype. Each radar plot summarizes the distinctive nutrient intake patterns extracted by SHAP analysis for each enterotype (Types A–E). The nutrients shown in each plot correspond to features with high SHAP signal frequency (SigFreq ≥ 0.5), indicating the most strongly contributing features to the classification of each type. For each nutrient, the z-score-standardized mean intake per 1,000 kcal was plotted for the corresponding enterotype (red) and all other enterotypes combined (blue). Values near the outer edge (positive z-scores) indicated a relatively higher intake than the cohort average, whereas values near the center (negative z-scores) indicated lower intake.

Based on SHAP-derived feature selection criteria (SigFreq ≥ 0.2, or SigFreq ≥ 0.1 together with RankFreq within the top 10 features for each enterotype), 15, 15, 7, 5, and 11 nutrients were prioritized as candidate features for Types A, B, C, D, and E, respectively (Figure 5 and Supplementary Figure 4). In Type A, the mean intake of 13 prioritized nutrients was higher than that in other enterotypes (Tables 2 and 3), indicating a consistently elevated nutrient intake profile. In contrast, in Type B, only 4 of 15 nutrients such as manganese and vitamin C showed a higher mean intake relative to the other types, whereas the remaining nutrients were lower. Type C was characterized by a lower intake of all prioritized nutrients. In Types D, fewer candidate nutrients were prioritized and mean intake of most of them were lower relative to other types. Similar to Type B, Type E showed a mixed nutritional profile, with relatively few prioritized nutrients exhibiting higher intake levels than the other enterotypes.

**Figure 6.**
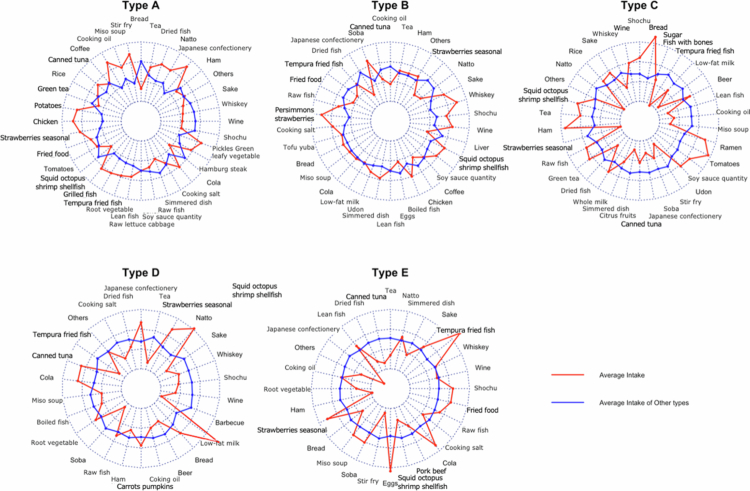
Radar plots of SHAP-extracted food-group intake profiles by enterotype. Each radar plot illustrates the characteristic food-group intake patterns extracted by SHAP analysis for each enterotype (Types A–E). Food items shown in each plot represent features with high SHAP signal frequency (SigFreq ≥ 0.7), indicating those that contributed most strongly to the classification of each type at the food-group level. See the legend of Figure 5 for details on the analytical procedure and interpretation of the z-scores.

In the food-group-based analyzes, more features were prioritized compared with nutrient-based analyzes. The number of Food groups prioritized were 36, 34, 34, 26, and 28 for Types A, B, C, D, and E, respectively (Figure 6 and Supplementary Figure 5). Among these, 11, 8, 7, 5, and 3 food groups were unique to Types A, B, C, D, and E, respectively (Tables 2 and 3).

**Table 2.** SHAP-extracted nutrients and food-group features commonly found between enterotypes.

Type A		Type B		Type C		Type D		Type E		
High	Low	High	Low	High	Low	High	Low	High	Low	
		Salt equivalent				Animal protein		Animal protein		Type A
		Vitamin C				Calcium		Vitamin C		
								Vitamin K		
		Black tea/oolong tea	Coffee	Black tea/oolong tea	Simmered dish	Canned tuna	Ham	Cooking oil	Bread	
		Cooking salt		Rice	Whiskey	Japanese confectionery	Sake	Fried food	Ham	
		Dried fish		Tomatoes		Natto	Shochu	Lean fish	Simmered dish	
		Miso soup				Root vegetables	Whiskey	Miso soup	Squid/octopus/shrimp/shellfish	
						Strawberries seasonal	Wine	Natto	Wine	
								Strawberries seasonal		
								Tempura fried fish		
					Animal protein		Vitamin B12	Vitamin C	*β*-Carotene	Type B
					Calcium				Copper	
					Copper				Plant protein	
					Plant protein				*δ*-Tocopherol	
					Vitamin B12				Vitamin B12	
				Black tea/oolong tea	Canned tuna	Bread	Cooking oil	Cola	Canned tuna	
				Bread	Cooking oil	Cola	Tempura fried fish	Miso soup	Japanese confectionery	
				Ham	Japanese confectionery	Raw fish		Raw fish	Stir fry	
				Raw fish	Lean fish			Sake		
				Sake	Low fat milk			Shochu		
				Shochu	Natto			Soba		
				Soy sauce quantity	Strawberries seasonal			Whiskey		
				Squid/octopus/shrimp/shellfish	Tempura fried fish					
				Udon						
				Wine						
							Vitamin C		Vitamin B12	Type C
							Vitamin B12		Plant protein	
									Copper	
						Bread	Cooking oil	Raw fish	Canned tuna	
						Raw fish	Dried fish	Sake	Dried fish	
							Miso soup	Shochu	Japanese confectionery	
							Soba		Simmered dish	
							Tempura fried fish			
							Whiskey			
								Animal protein	Vitamin B12	Type D
								Cola	Black tea/oolong tea	
								Natto	Cooking salt	
								Raw fish	Dried fish	
								Strawberries seasonal	Ham	
									Wine	

**Table 3.** SHAP-extracted nutrients and food-group features uniquely found in each enterotype.

Type A		Type B		Type C		Type D		Type E	
High	Low	High	Low	High	Low	High	Low	High	Low
Animal Fat	C12:0(S)	Alcohol	Carbohydrate		*n*-3 Fatty acids		Cryptoxanthin	Retinol equivalent	*α*-Tocopherol
Copper	Sucrose	Manganese	*α*-Carotene						Daidzein
Daidzein			Folic acid						
*n*-3 Fatty acids			Vitamin K						
Plant Fat									
Plant protein									
Vitamin B12									
*δ*-Tocopherol									
Chicken	Cola	Boiled fish	Chicken	Beer	Citrus fruit seasonal	Carrots pumpkins	Barbecue	Egg	Root Vegetables
Grilled fish	Green tea	Persimmons/strawberries	Eggs	Green tea	Fish with bones	Low-fat milk	Beer		Pork and Beef
Hamburg steak	Raw fish	Simmered dish	Fried food	Whole milk	Sugar		Boiled fish		
Potatoes	Soy sauce	Tofu/yuba	Liver	Ramen					
Raw lettuce cabbage									
Stir fry									
Pickles green leafy vegetables									

In both nutrient- and food-group-based analyzes, the LD1 scores obtained from LDA differed significantly between each enterotype and the remaining types (all *p* < 0.05), indicating that the SHAP-selected features were sufficient to distinguish enterotype-specific dietary patterns. The corresponding effect sizes (r) ranged from small to moderate and were mostly higher in the diet-based analyzes (Supplementary Figures 6 and 7).

### Correlations between nutrient intake and the abundance of bacterial taxonomy

**Figure 7.**
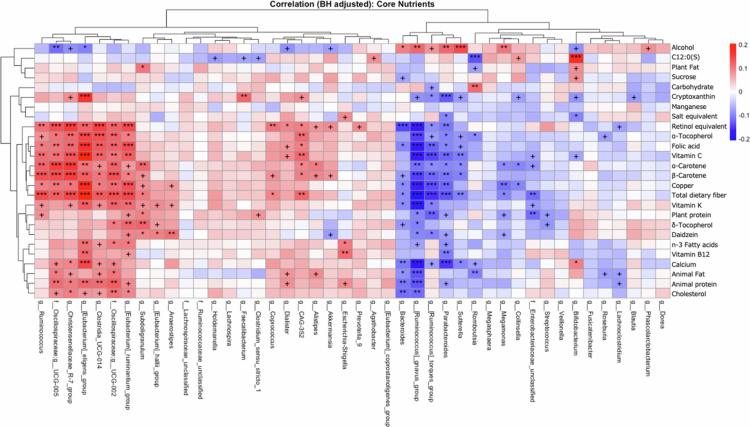
Heatmaps showing Spearman’s correlations between nutrient intakes and bacterial genera. Each heatmap represents the correlation matrices between gut bacterial genera (columns) and nutrients grouped by category (rows). Color intensity indicates the strength and direction of the correlation (red: positive; blue: negative). Asterisks denote significance levels based on BH-adjusted *P*-values (*P < 0.1, **P < 0.05, and ***P < 0.01). Nutrients were normalized per 1,000 kcal, and 26 representative nutrients were selected.

**Figure 8.**
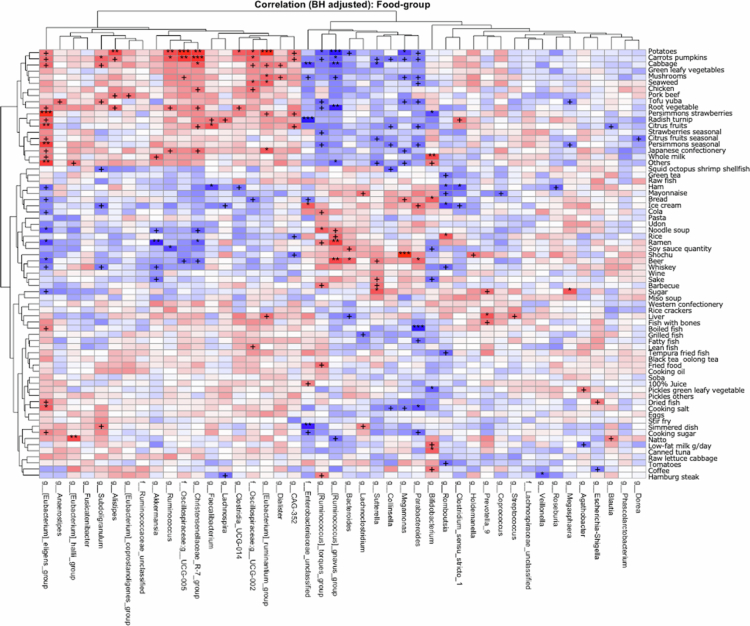
Heatmaps showing Spearman’s correlations between intakes of food-group and bacterial genera. See the legend of Figure 7 for details of the analytical procedure and interpretation.

Many significant correlations were observed between the relative abundances of the major bacterial taxa and nutrient or food-group intake, as shown in Figures 7 and 8 and Supplementary Figures 8–12. To maintain consistency in the feature selection process, we specifically described correlations involving SHAP-prioritized features that were identified as contributing to enterotype classification (Figures 7 and 8).

We evaluated the association between each enterotype and gut microbiota in Figure 2 to extract representative microbial profiles characterizing each enterotype. We assessed the relationship between the extracted microbial composition and various nutrients and food groups. For Type A, the key taxa *Oscillospiraceae* UCG-002 and UCG-005 showed strong positive correlations with SHAP-prioritized nutrients, including animal protein, animal fat, vitamin C and copper, while *Oscillospiraceae* UCG-002 also correlated positively with vitamin K and *δ*-tocopherol. Both taxa correlated positively with the intake of potatoes, and *Oscillospiraceae* UCG-002 correlated positively with the intake of chicken. In contrast, the *Eubacterium coprostanoligenes* group, another key taxon in Type A showed no significant correlation with any SHAP-prioritized features.

The key taxon of Type B, *Bacteroides*, displayed negative correlations with several SHAP-prioritized nutrients (animal protein, copper, *δ*-tocopherol, vitamin K, calcium, and folic acid), while positively correlated with soy source quantity.

In Type C, *Megamonas* abundance was negatively with plant protein and copper. It also correlated positively with shochu and beer consumption but negatively with Japanese confectioneries. *Akkermansia*, another key taxon, showed no correlations with SHAP-prioritized nutrients while strong positive correlations with whole milk intake and was inversely associated with ramen and whiskey consumption.

The abundance of *Bifidobacterium* in Type D was negatively correlated with vitamin C, and cryptoxanthin intake and positively associated with calcium intake. At the food-group level, this taxon was positively correlated with bread, low-fat milk, and canned tuna and negatively correlated with sake.

Finally, the key taxa in Type E, *Prevotella* 9 and *Dialister*, showed distinct associations. *Prevotella* 9 was positively correlated with retinol equivalents, whereas *Dialister* correlated positively with vitamin C, retinol equivalent, animal fat, and animal protein. No significant food-level correlations were observed for *Prevotella* 9 and *Dialister*.

## Discussion

We investigated the relationship between enterotypes and habitual dietary intake in a large cohort of older Japanese individuals. By reassigning bacterial taxonomy using the SILVA 138 database, we revealed new enterotype features beyond those described in the outdated Greengenes database. Furthermore, the application of SHAP values derived from the XGBoost classifiers enabled us to screen combinations of nutrients and food groups that can associated with enterotype structure. These approaches provided a more comprehensive understanding of how diet may shape the gut microbiota structure in this population.

This study had several strengths. First, the relatively large sample size (*n* = 770) provided sufficient statistical power and robustness to evaluate the dietary associations with enterotypes. Second, the updated taxonomy enabled a higher-resolution characterization of genus-level features within enterotypes. For example, while our previous study identified an enrichment of *Ruminococcaceae* in Type A,^5^ the current analysis using the SILVA 138 database clarified that this enrichment was largely driven by specific genera such as *Oscillospiraceae* UCG-002, *Oscillospiraceae* UCG-005, and the *Eubacterium coprostanoligenes* group. Additionally, the enrichment of *Prevotella* 9, which includes *Prevotella copri*, was revealed as a defining feature of Type E. Third, the introduction of nutrient intake scores based on the Japanese Dietary Reference Intakes allowed us to evaluate dietary adequacy more comprehensively, highlighting not only excesses and deficiencies but also enterotype-specific imbalances. Finally, the combined use of SHAP and XGBoost enabled exploratory prioritization of dietary features potentially associated with enterotype classification while accounting for the combined effects of multiple dietary variables. Rather than relying solely on univariate comparisons, this approach allowed us to identify reproducibly prioritized nutrients and food groups across repeated model iterations. In particular, our findings confirmed that insufficient intake of dietary fiber, calcium, and several vitamins, together with excessive salt intake, was a pervasive issue across all enterotypes. Despite this common background, each enterotype exhibited a distinct dietary pattern.

Simple comparisons of nutrient and food-group intake as well as correlations between key taxa and dietary variables suggested an association between diet and enterotype. The SHAP-based XGBoost analyzes further indicated that combinations of multiple dietary variables may collectively contribute to enterotype-associated dietary tendencies, although the predictive performance was modest. Compared with nutrient-based analyzes, food-group-based analyzes generally showed clearer separation and larger effect sizes in LDA, suggesting that habitual dietary patterns and food combinations may better capture enterotype-associated ecological differences than individual nutrients alone.

LDA demonstrated that each enterotype could be distinguished by using multiple SHAP-prioritized features. Nevertheless, the effect sizes of LD1 were small to moderate, suggesting that diet alone does not fully explain enterotype composition. Other factors, such as medication use, which is known to strongly affect the gut microbiota,^18^ as well as age and BMI, both of which differed among enterotypes in this study, are also likely to contribute to enterotype formation.^19^ ^,^ ^20^

The dietary patterns predicted based on the SHAP-prioritized features, especially those uniquely identified in each type, were as follows: Type A was characterized by a generally higher intake of multiple nutrients, suggesting a surplus of substrates available for fermentation, which may contribute to the absence of a single dominant taxon. Dietary fiber and fractions of dietary protein and fat can reach the colon and serve as substrates for gut microbiota.^21^ ^,^ ^22^ Type B showed a relatively balanced intake pattern with fewer extreme deviations, consistent with the Bacteroide-dominated microbiota. Because approximately half of Japanese individuals lack detectable *Prevotella* (based on data from about 2,000 Japanese individuals; this study and Takagi et al., ^5^ such balanced nutrient profiles may predispose non-*Prevotella* carriers to the Type B configuration. In contrast, Type C was characterized by lower overall nutrient fulfillment, with carbohydrate-rich diets such as rice, bread and noodles predominating. Type D shared similarities with Type A but showed associations with low-fat milk, which were strongly correlated with the abundance of *Bifidobacterium*. Cow milk has been reported to have bifidogenic properties that promote increases in *Bifidobacterium.* ^23^ ^,^ ^24^ However, our previous study indicated a strong association between this enterotype and gastrointestinal diseases, particularly IBD.^5^ Thus, disease-related effects on microbial composition cannot be ruled out. Type E was notable for a higher intake of retinol equivalents, likely derived from egg and fish (Lean fish, raw fish and tempura fried fish), which aligned with the enrichment of *Prevotella* 9 and *Dialister*. In addition, less intake of pork and beef was suggested in Type E. Previous studies have reported an inverse association between red meat consumption and *Prevotella* abundance,^25^ whereas retinol intake has been positively associated.^26^ Nevertheless, it should be acknowledged that the BDHQ used in this study had limited ability to capture grain consumption, a well-known substrate for *Prevotella,* ^27^ which may partly explain the dietary associations observed for Type E.

This study had several limitations. Although enterotypes were treated as discrete categories in the present study, recent studies have suggested that gut microbiota variation may also be represented as continuous gradients rather than strictly separated clusters.^28^ Therefore, enterotypes should be interpreted as a pragmatic framework for summarizing major microbiota configurations and exploring microbiota-associated phenotypes rather than as completely discrete biological entities. The population was restricted to older residents of the Kyotango region, which is characterized by longevity with lower levels of frailty and distinctive dietary practices,^29^ ^,^ ^30^ and thus the findings may not be generalizable to younger or more diverse populations. Second, detailed information regarding medication use was not systematically collected in this study. Since various medications, including antibiotics, proton pump inhibitors, metformin, and other commonly prescribed drugs, may influence gut microbiota composition and enterotype formation, residual confounding by medication use cannot be excluded. Third, the BDHQ, although validated in Japanese cohorts, may not adequately capture certain food groups, such as grains, which are important for interpreting associations with the gut microbiota, particularly *Prevotella*. The cross-sectional design also precludes causal inference. Finally, the use of 16S rRNA gene sequencing limited the ability to directly assess microbial function.

Future studies should aim to validate these findings across broader age groups and populations, ideally using longitudinal and interventional designs, to clarify causal relationships. Multi-omics approaches, including shotgun metagenomics and metabolomics, are essential for revealing the functional implications of diet–enterotype associations. Additionally, improved dietary assessment methods that more accurately capture culturally relevant food groups will enhance our ability to interpret microbiota–diet interactions, particularly for *Prevotella*-associated enterotypes.

In conclusion, this study demonstrates that Japanese gut microbiota enterotypes in older adults are associated with distinct but partially overlapping combinations of habitual nutrient and food-group intake, rather than with single dietary components. By integrating updated taxonomic annotation with machine learning–based interpretation, we identified prioritized dietary features potentially associated with enterotype structure. Although dietary patterns alone did not fully explain enterotype composition, they contributed substantially to discrimination. These findings provide a foundation for developing enterotype-aware dietary strategies and highlight the potential of personalized nutritional approaches to support healthy aging by modulating gut microbiota.

## Supplementary Material

Supplementary Material Suppl_Fig3

Supplementary Material Suppl_Fig4

Supplementary Material Suppl_Fig12

Supplementary Material Suppl_Fig7

Supplementary Material Suppl_Fig9

Supplementary Material Suppl_Fig6

Supplementary Material Suppl_Fig1

Supplementary Material Suppl_Fig2

Supplementary Material Suppl_Fig11

Supplementary Material Suppl_Fig8

Supplementary Material Suppl_Fig10

Supplementary Material Suppl_Fig5

Supplementary_Figures_tables.docx Supplemental Material

## Data Availability

Raw sequencing data have been deposited in the NCBI Sequence Read Archive (SRA) under BioProject ID PRJNA1440982. The data are currently under controlled access and will be made publicly available upon publication of this article. During peer review, the data are accessible via the following private link: https://dataview.ncbi.nlm.nih.gov/object/PRJNA1440982?reviewer=34krs7kauassc617o1nk4edt22.
