# Supplementary figures and images for: Assessment of nutrient intake by gut microbiota enterotype in Japanese subjects
Source: Gut Microbes Rep. 2026 Jul 30;3(1):2705623. doi: 10.1080/29933935.2026.2705623 (PMC13432868; doi:10.1080/29933935.2026.2705623)

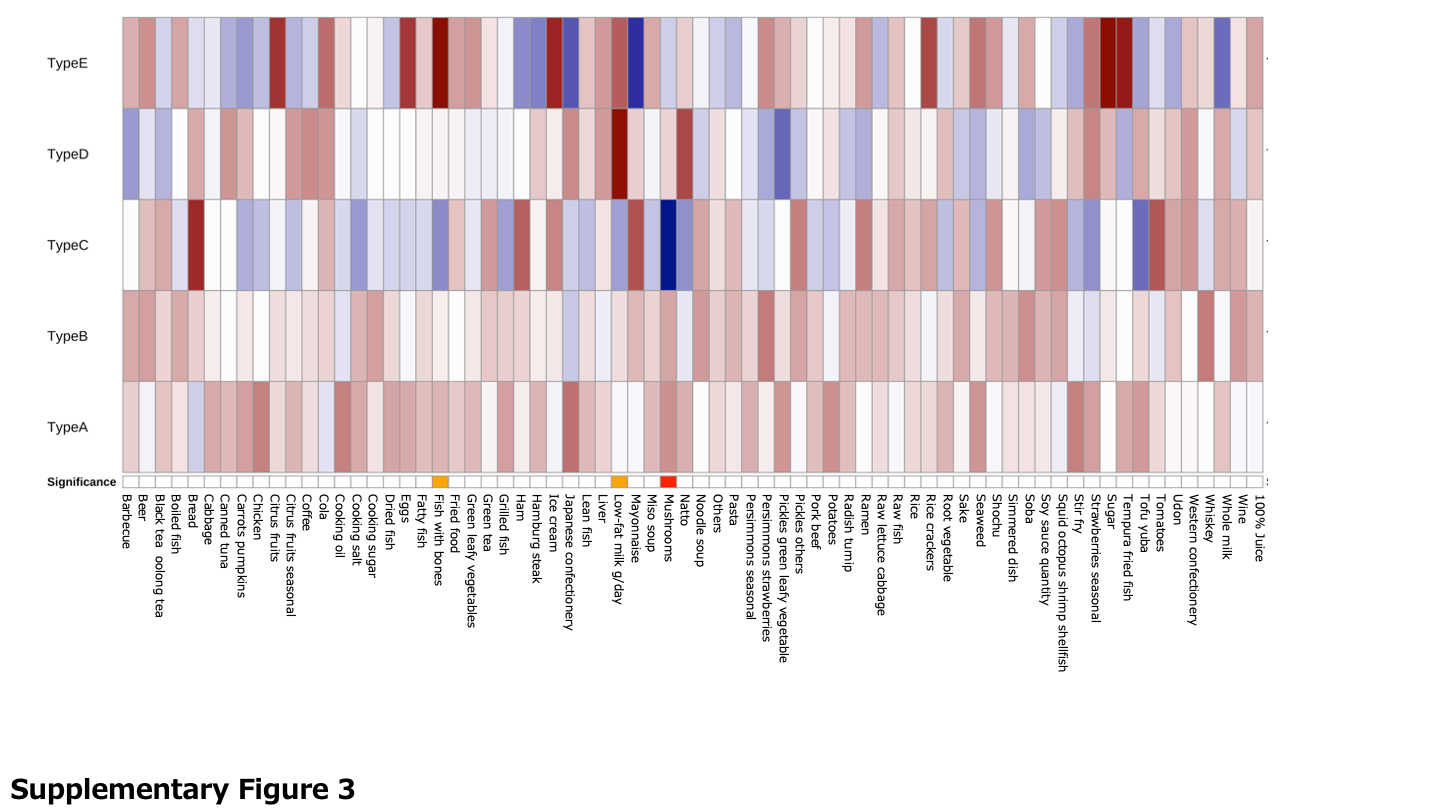

Supplement: Supplementary Material — Suppl_Fig3 [file KGMR_A_2705623_SM4474.tif]

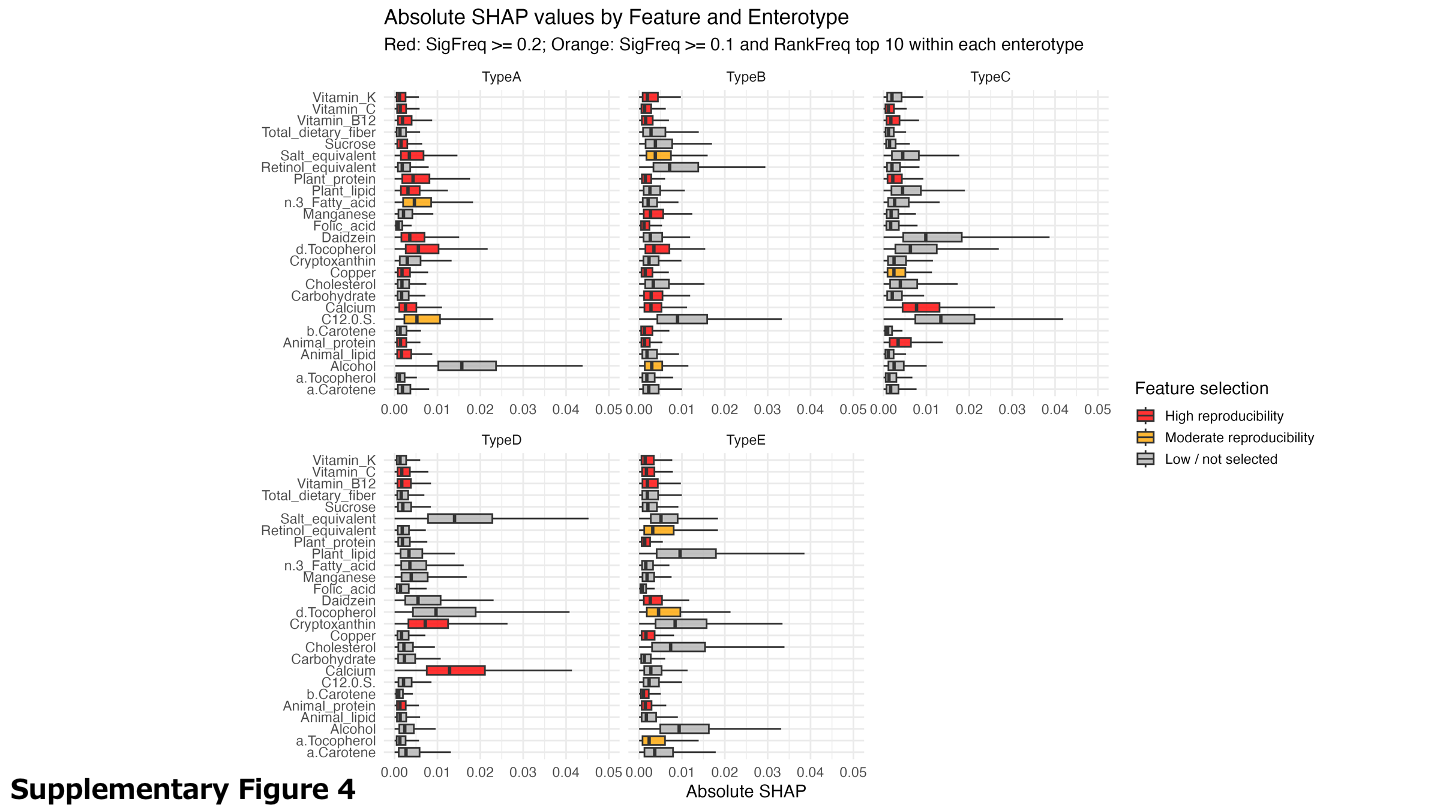

Supplement: Supplementary Material — Suppl_Fig4 [file KGMR_A_2705623_SM4473.tif]

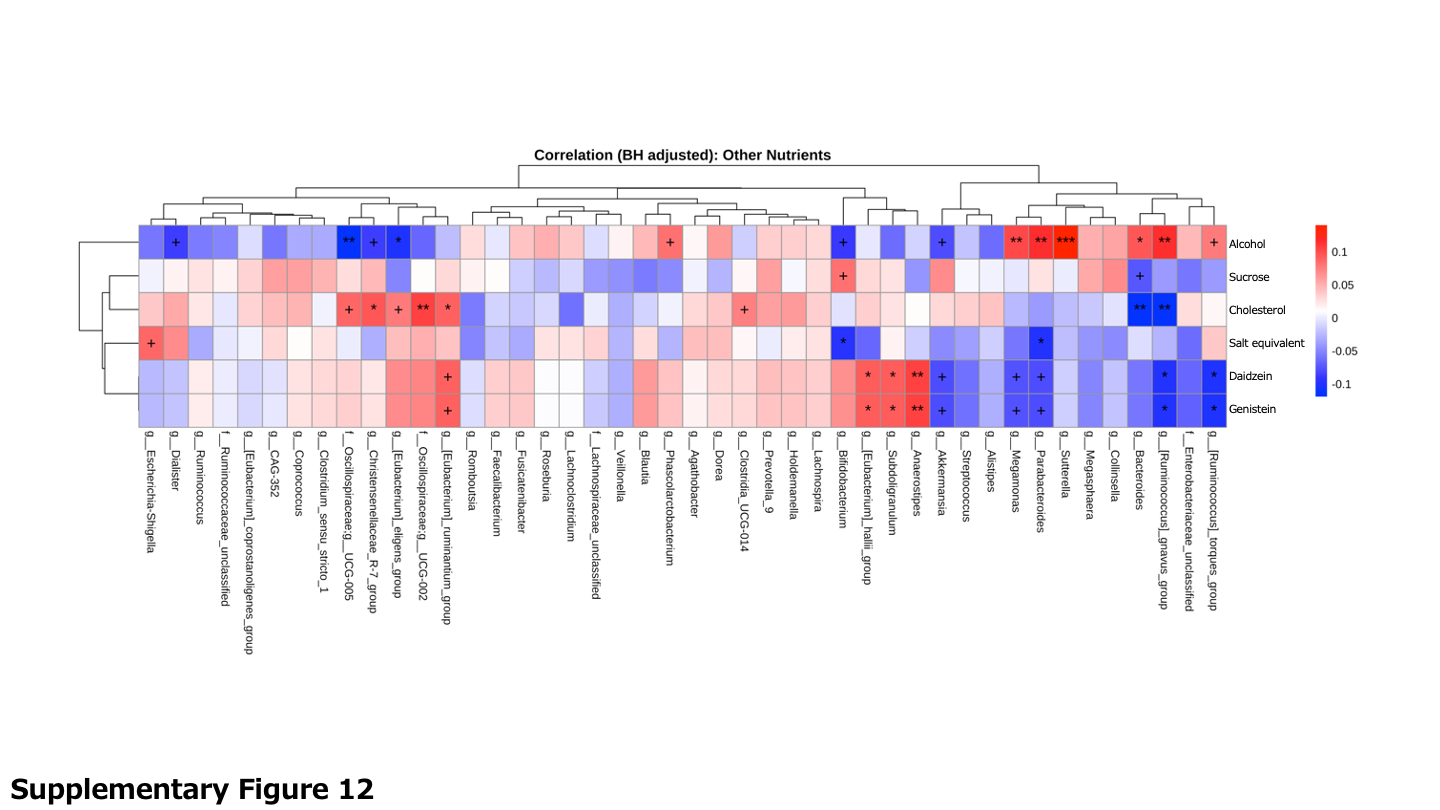

Supplement: Supplementary Material — Suppl_Fig12 [file KGMR_A_2705623_SM4472.tif]

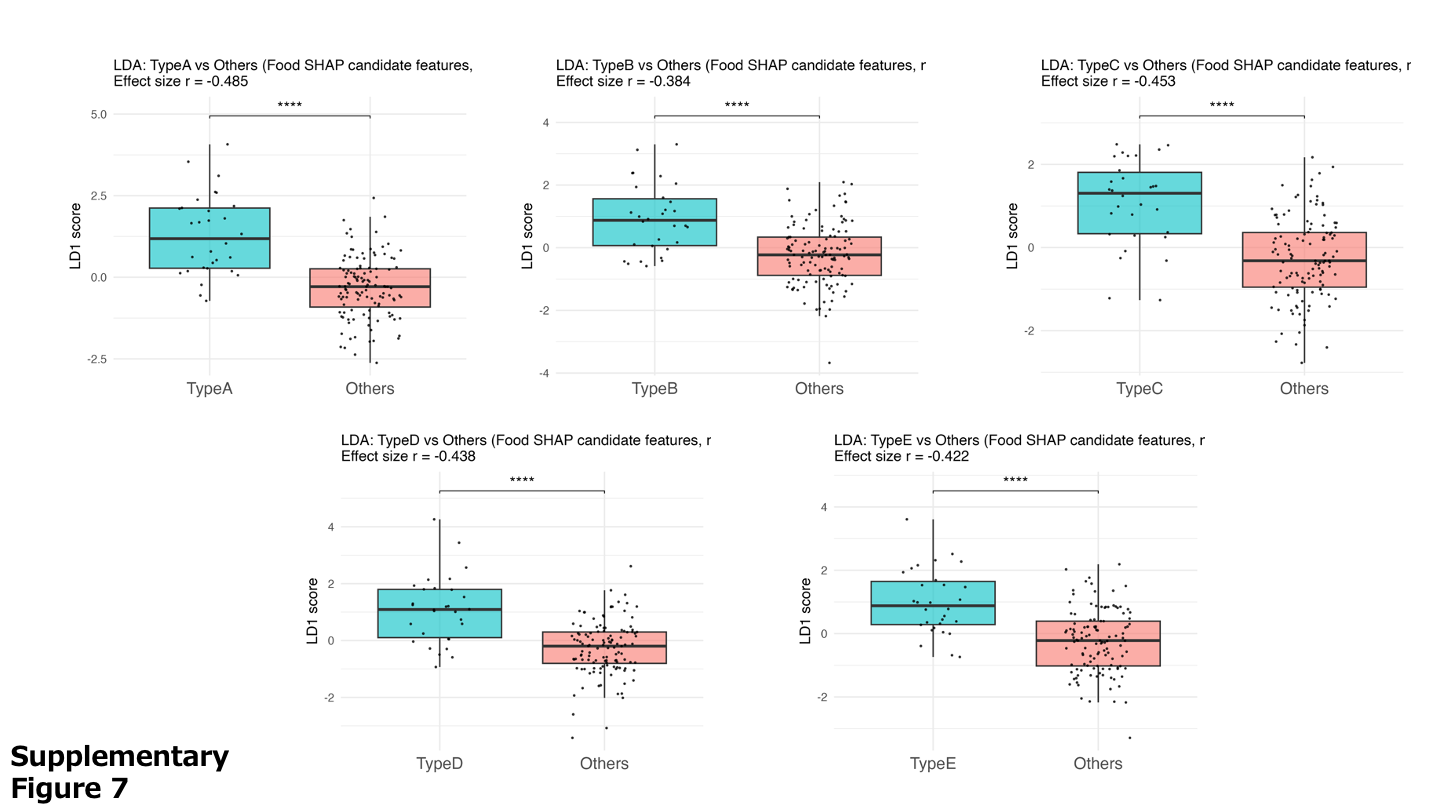

Supplement: Supplementary Material — Suppl_Fig7 [file KGMR_A_2705623_SM4470.tif]

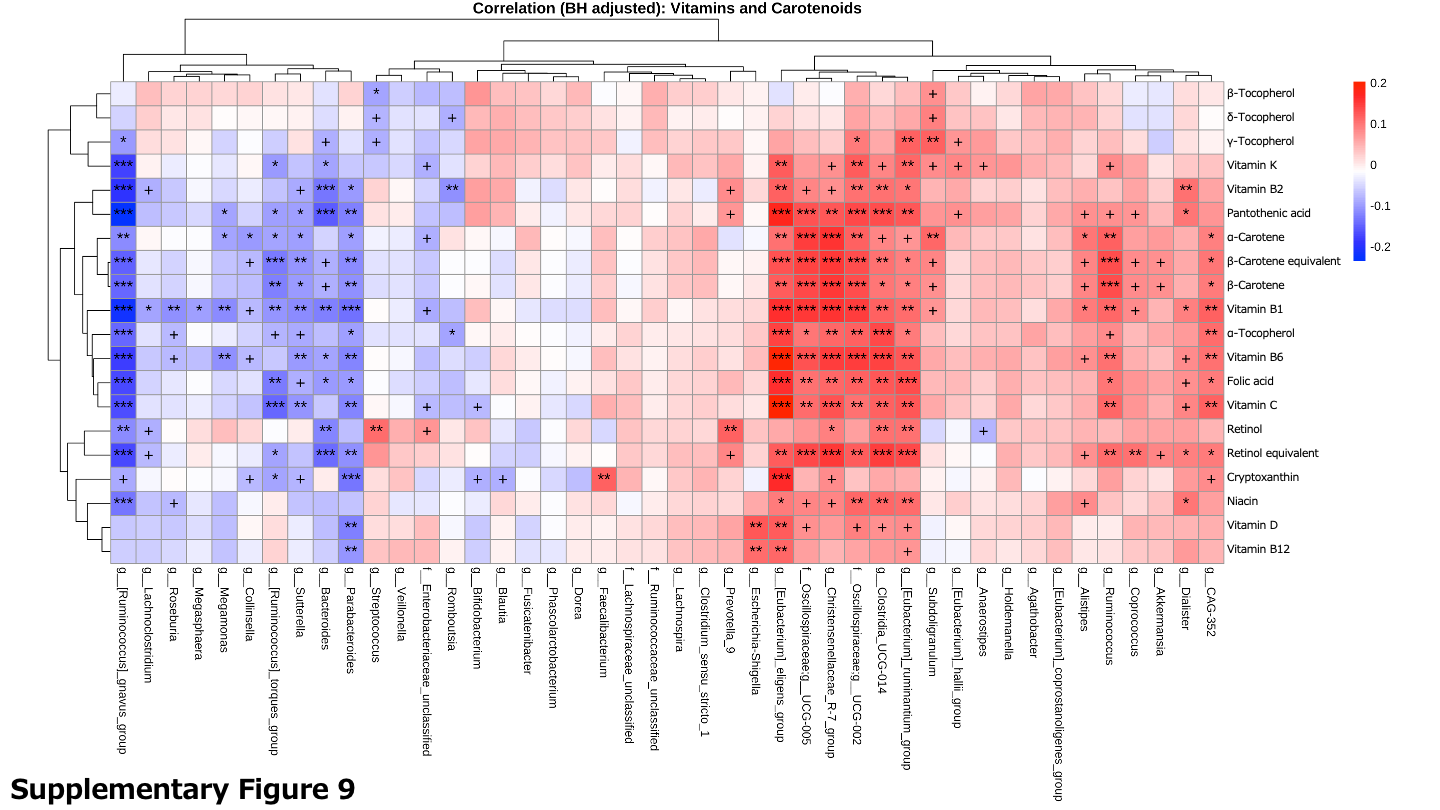

Supplement: Supplementary Material — Suppl_Fig9 [file KGMR_A_2705623_SM4469.tif]

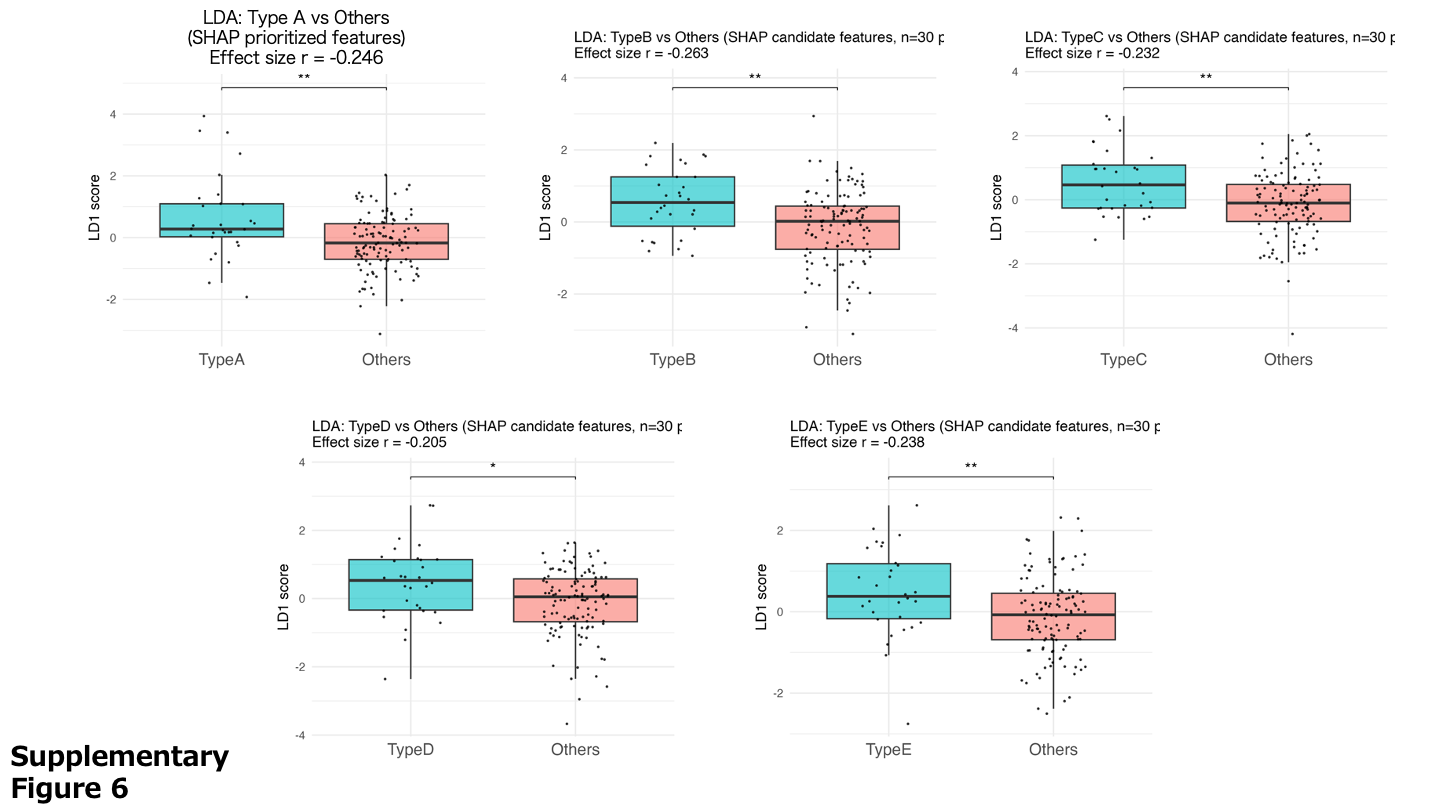

Supplement: Supplementary Material — Suppl_Fig6 [file KGMR_A_2705623_SM4468.tif]

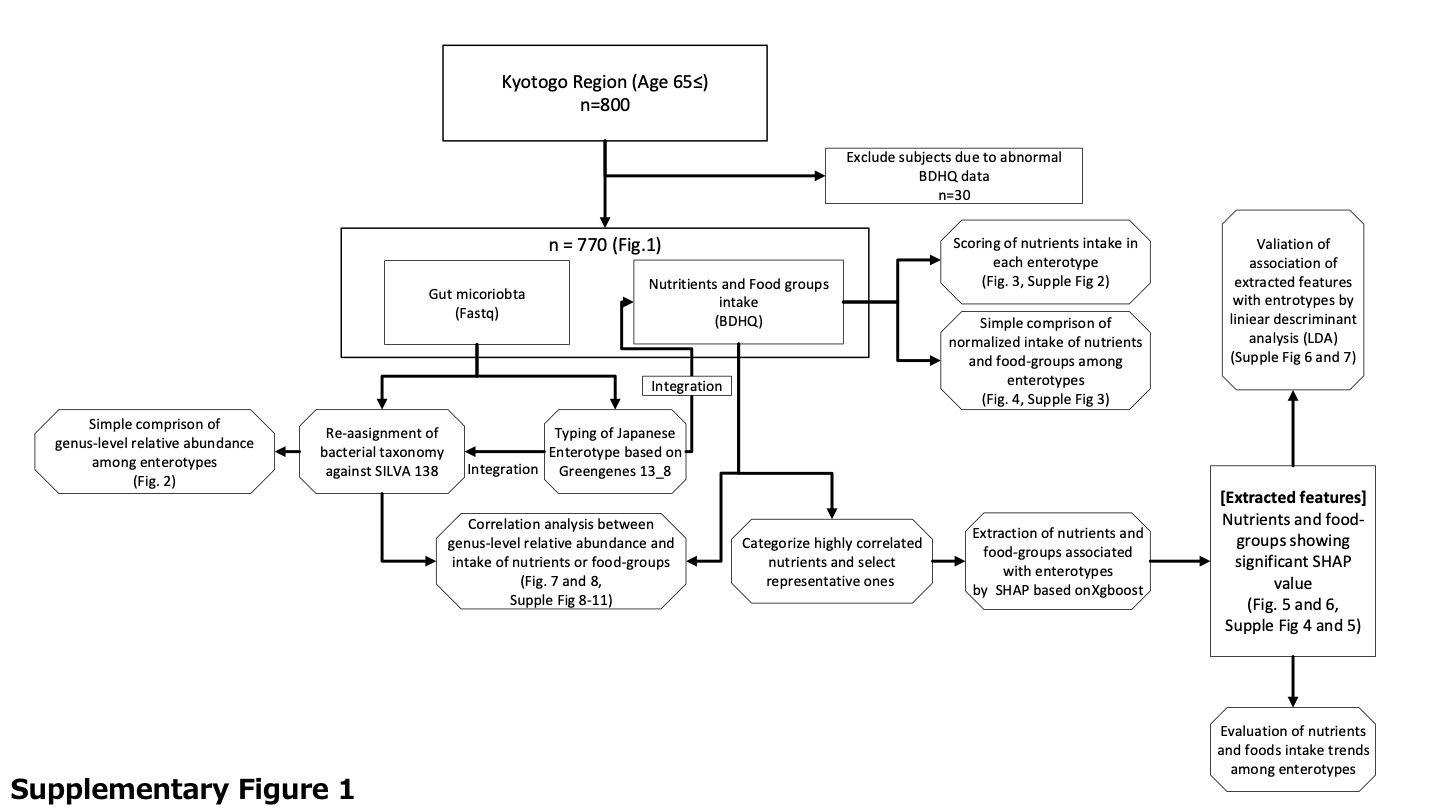

Supplement: Supplementary Material — Suppl_Fig1 [file KGMR_A_2705623_SM4467.tif]

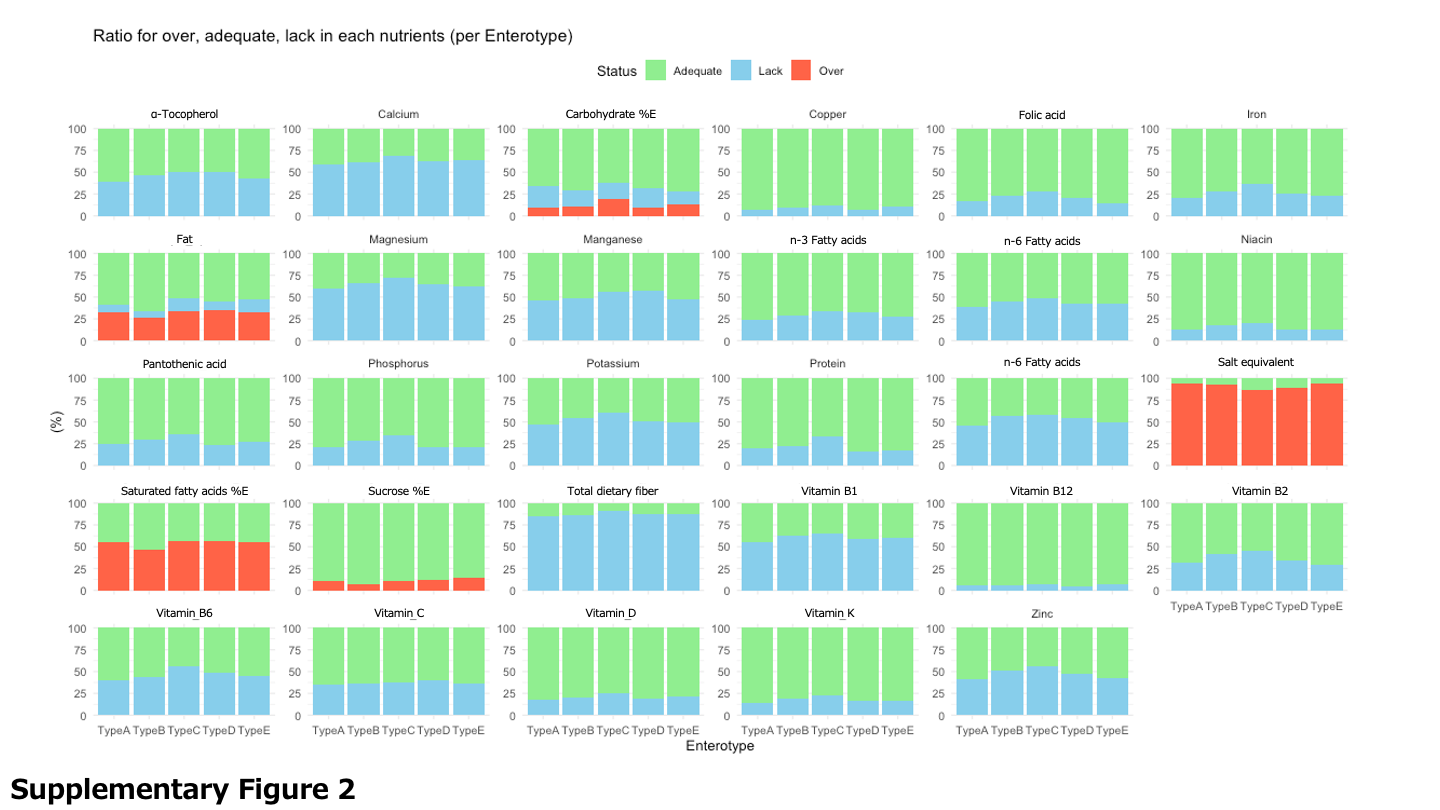

Supplement: Supplementary Material — Suppl_Fig2 [file KGMR_A_2705623_SM4466.tif]

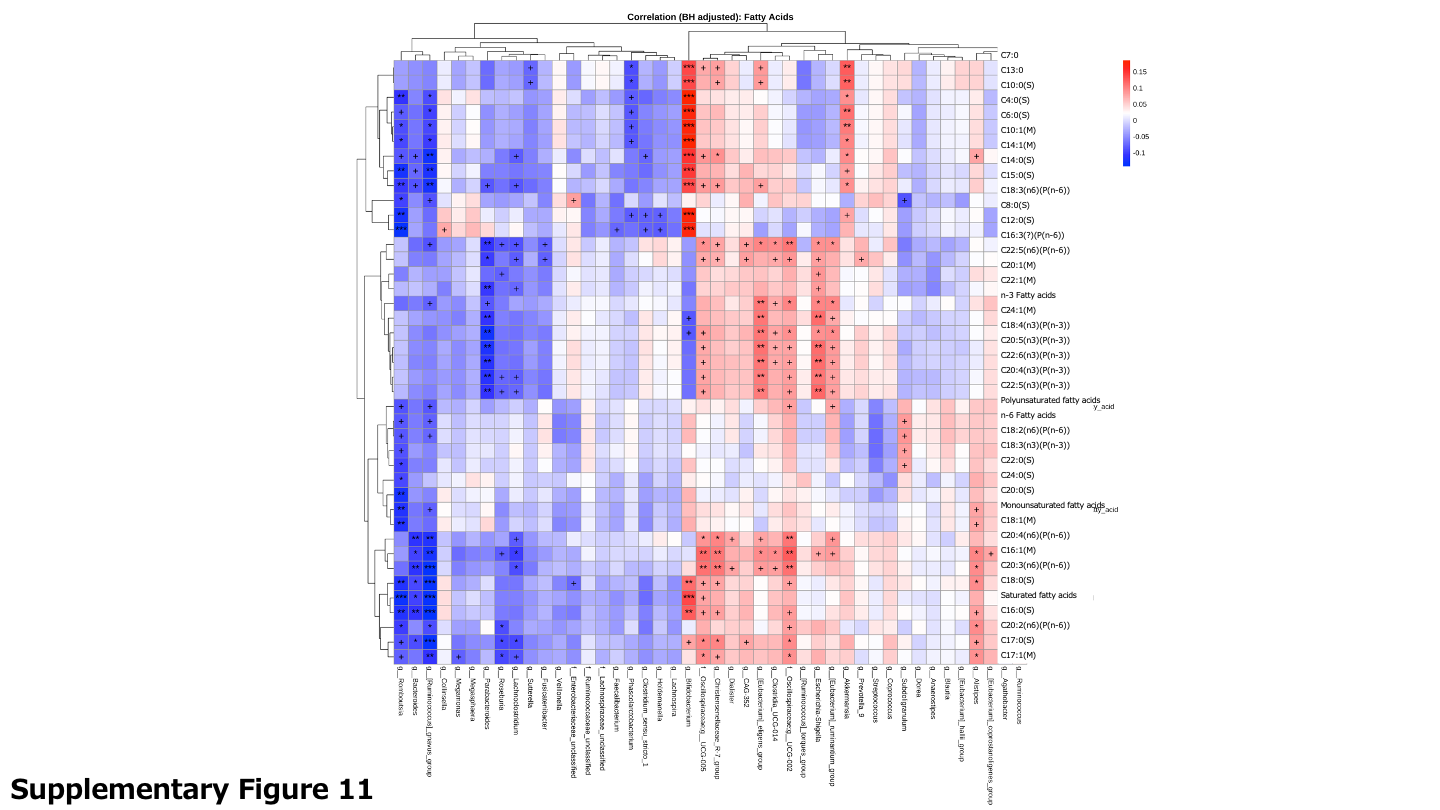

Supplement: Supplementary Material — Suppl_Fig11 [file KGMR_A_2705623_SM4465.tif]

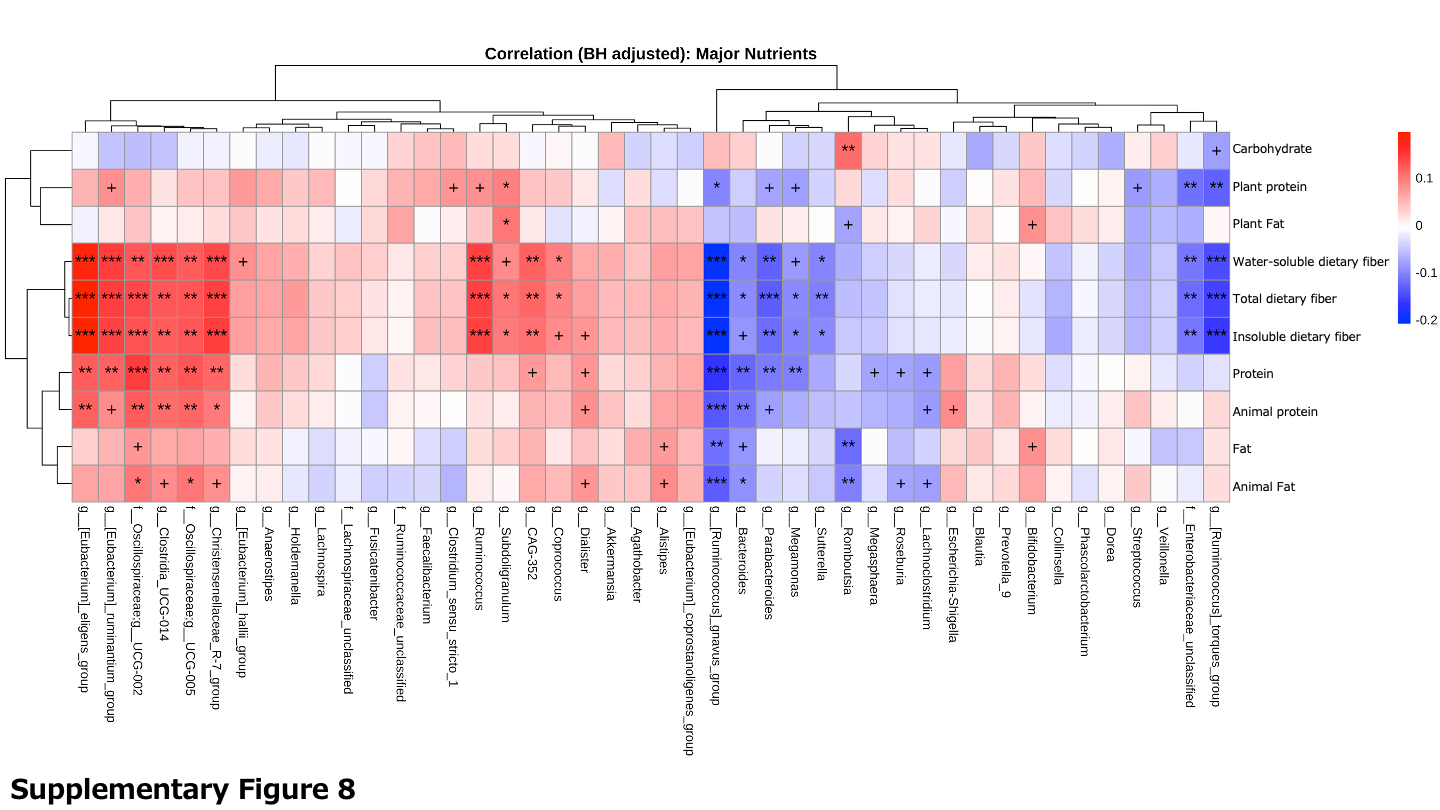

Supplement: Supplementary Material — Suppl_Fig8 [file KGMR_A_2705623_SM4464.tif]

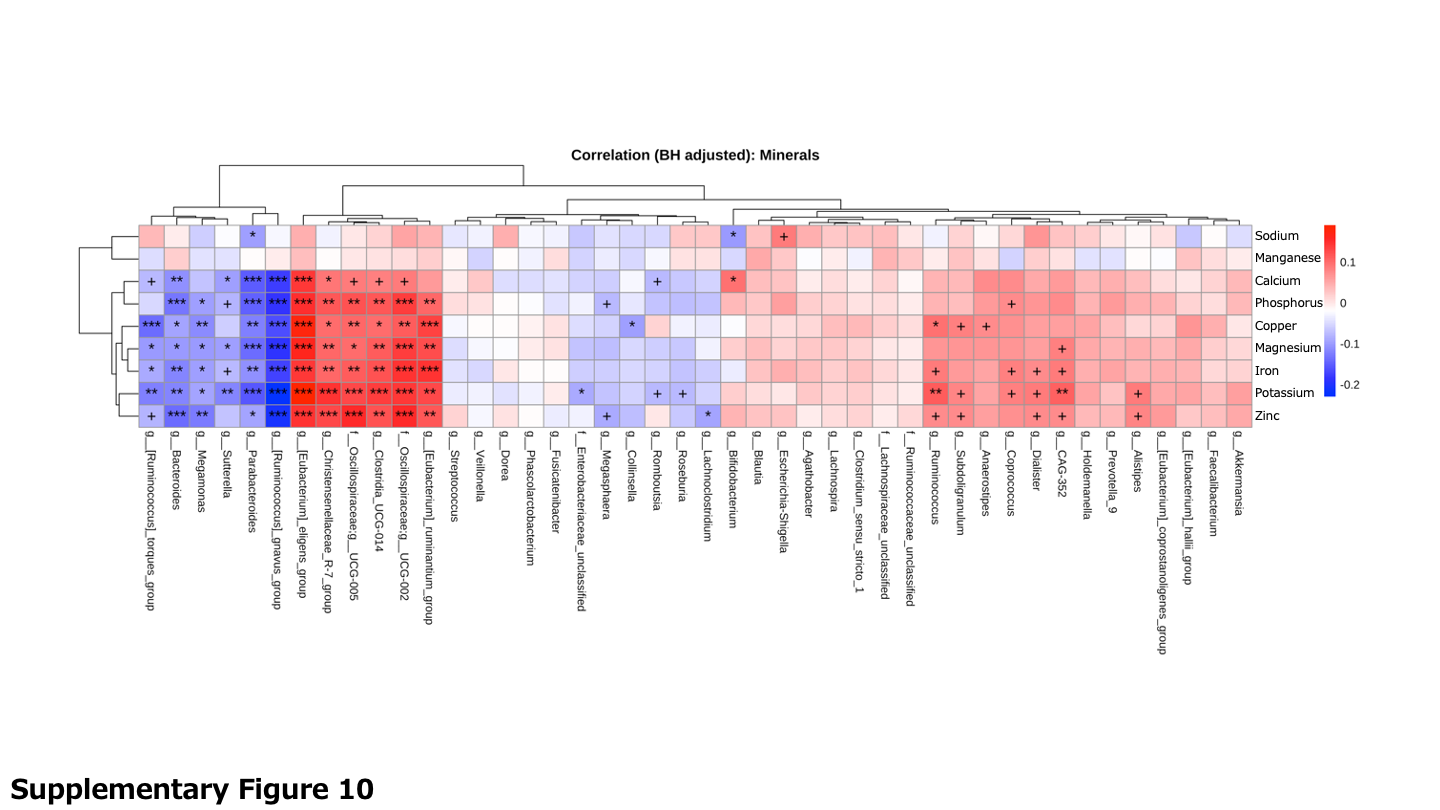

Supplement: Supplementary Material — Suppl_Fig10 [file KGMR_A_2705623_SM4463.tif]

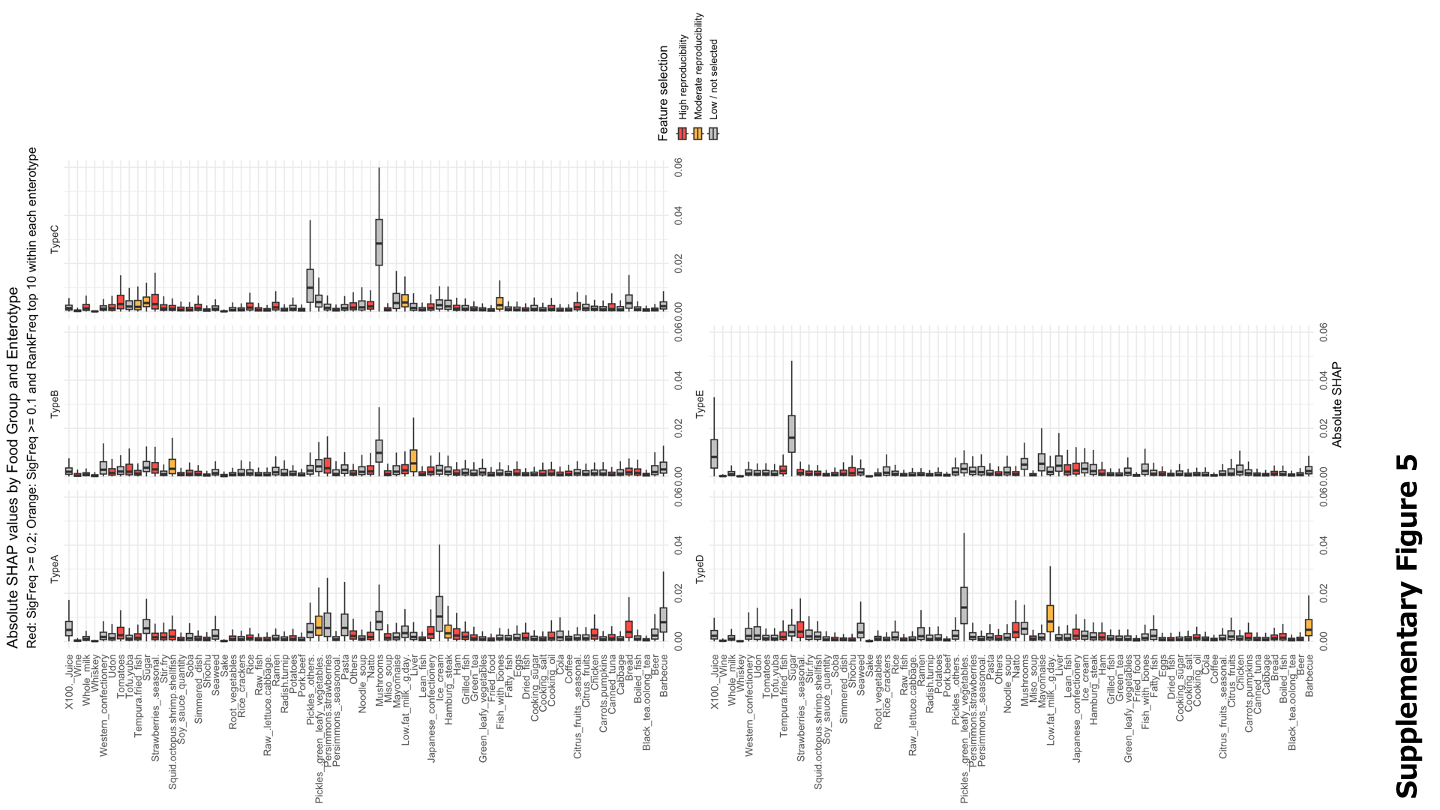

Supplement: Supplementary Material — Suppl_Fig5 [file KGMR_A_2705623_SM4462.tif]
