# Supplementary material for: Assessment of nutrient intake by gut microbiota enterotype in Japanese subjects
Source: Gut Microbes Rep. 2026 Jul 30;3(1):2705623. doi: 10.1080/29933935.2026.2705623 (PMC13432868; doi:10.1080/29933935.2026.2705623)
Supplement: Supplementary_Figures_tables.docx — Supplemental Material [file KGMR_A_2705623_SM4999.docx]

**Supplementary Figure 1**

Flow of the analyses performed in this study.

**Supplementary Figure 2**

Proportion of participants with nutrient intakes classified as “Lack,” “Adequate,” or “Over” in each enterotype.

For each nutrient, participants were categorized based on the Japanese Dietary Reference Intakes (2025 edition) as having insufficient (“Lack”), adequate (“Adequate”), or excessive (“Over”) intake relative to the reference range. The bars represent the percentage of participants within each enterotype who fell into these categories for each nutrient.

Sky blue, light green, and tomato colors indicate “Lack,” “Adequate,” and “Over,” respectively.

**Supplementary Figure 3**

Mean standardized (z-score) food-group intakes per 1,000 kcal across enterotypes.

This figure extends the analysis shown in Figure 4 by replacing the nutrients with individual food groups.

The heatmap displays the mean z-scores of food-group intake normalized per 1,000 kcal for each enterotype (Types A–E).

Rows represent enterotypes, and columns represent food groups. For each food-group, the intake values were standardized within the cohort (z-score transformation), and the mean values were calculated by enterotype.

Statistical differences among enterotypes were assessed as described in Figure 2.

For row-side annotations and color intensity, see Figure 4.

**Supplementary Figure 4**

Feature importance and reproducibility of SHAP-derived nutrient predictors across enterotypes.

Boxplots show the distribution of the absolute SHAP values for each nutrient feature across 50 repeated XGBoost models. Each panel corresponds to one enterotype (Types A–E).

Red, orange, and gray bars represent features with high (SigFreq ≥ 0.7), moderate (0.5 ≤ SigFreq < 0.7), and low (SigFreq < 0.5) signal frequencies, respectively, indicating the reproducibility of feature importance across model repetitions.

Higher absolute SHAP values indicate stronger contributions of the corresponding nutrients to the prediction of each enterotype.

**Supplementary Figure 5**

Feature importance and reproducibility of SHAP-derived food-group predictors across enterotypes.

Boxplots show the distribution of the absolute SHAP values for each food-group feature across the 50 repeated XGBoost models. Each panel corresponds to one enterotype (Types A–E). See the legend of Supplementary Figure 4 for details on the analytical procedure and interpretation.

**Supplementary Figure 6**

Linear discriminant analysis (LDA) distinguishing each enterotype from others based on SHAP-extracted nutrient features.

Each panel shows the LDA results comparing one enterotype (Type A–E) against all other types using nutrient variables with SHAP signal frequency ≥ 0.5.

LDA was performed using nutrient intake normalized per 1,000 kcal, with 30 randomly selected participants per enterotype. The LD1 axis represents the primary discriminant function that separates the target enterotype from the other enterotypes.

Boxplots display LD1 scores between groups, with statistical significance assessed using the Wilcoxon rank-sum test (BH-adjusted).

**Supplementary Figure 7**

Linear discriminant analysis (LDA) distinguishes each enterotype from others based on SHAP-extracted features of the food-group.

Each panel shows the LDA results comparing one enterotype (Types A–E) with the others, using food-group variables with SHAP signal frequency ≥ 0.7.

See the legend of Supplementary Figure 6 for details on the analytical procedure and interpretation.

**Supplementary Figure 8**

Heatmaps showing Spearman’s rank correlations between macronutrient intake assessed by BDHQ and bacterial genera.

See the legend of Figure 7 for details of the analytical procedure and interpretation.

**Supplementary Figure 9**

Heatmaps showing Spearman’s correlations between intakes of carotenoids/vitamins assessed by BDHQ and bacterial genera.

See the legend of Figure 7 for details of the analytical procedure and interpretation.

**Supplementary Figure 10**

Heatmaps showing Spearman’s rank correlations between mineral intake assessed by BDHQ and bacterial genera.

See the legend of Figure 7 for details of the analytical procedure and interpretation.

**Supplementary Figure 11**

Heatmaps showing Spearman’s correlations between intakes of fatty acids assessed by BDHQ and bacterial genera.

See the legend of Figure 7 for details of the analytical procedure and interpretation.

**Supplementary Figure 12**

Heatmaps showing Spearman’s rank correlations between intakes of other nutrients assessed by BDHQ and bacteria genera.

See the legend of Figure 7 for details of the analytical procedure and interpretation.
